# zelll: a fast, framework-free, and flexible implementation of the cell lists algorithm for the Rust programming language

**DOI:** 10.1093/bioadv/vbaf330

**Published:** 2026-01-02

**Authors:** Vincent Messow, Christian Höner zu Siederdissen, Michael Habeck

**Affiliations:** Microscopic Image Analysis Group, Jena University Hospital, 07747 Jena, Germany; Independent Researcher; Microscopic Image Analysis Group, Jena University Hospital, 07747 Jena, Germany; Max Planck Institute for Multidisciplinary Sciences, 37077 Göttingen, Germany

## Abstract

**Summary:**

The cell lists algorithm is widely used to compute pairwise particle interactions below a fixed cutoff distance in approximately linear time. Prominent molecular dynamics frameworks implementing cell lists variants assume pre-determined and densely populated simulation boxes suitable for e.g. all-atom simulations with explicit solvents. zelll implements a simple yet efficient variant of the cell lists algorithm that uses sparse storage for the underlying partitioning grid. This allows for applications with dynamic simulation boundaries and sparsely populated simulation space not strictly fitting into the scope of common molecular dynamics frameworks, such as many coarse-grained simulations. For this reason, zelll does not target specific frameworks.

**Availability and implementation:**

zelll is an open-source Rust library available under the MIT license at https://github.com/microscopic-image-analysis/zelll and https://crates.io/crates/zelll. Python bindings are available at https://pypi.org/project/zelll.

## 1 Introduction

When particle-based simulations compute pairwise interactions, it is usually necessary to enumerate all interacting particle pairs. In general, this is of $O(n^{2})$ runtime complexity for *n* particles. In certain applications, e.g. molecular dynamics (MD), interactions between distant particles are often assumed negligible. In such cases, the cell lists algorithm has been widely used for decades to enumerate all particle pairs closer than a certain cutoff distance $r_{c}$ in linear time (Quentrec and Brot 1973). We describe an implementation for the Rust programming language that benefits from language features but is otherwise closely following the basic cell lists algorithm. As a result, our implementation can be easily adjusted toward needs which may not be covered by commonly used MD frameworks. Specifically, zelll is primarily intended for settings where simulation space is potentially sparsely populated with structured, coarse-grained data, e.g. in chromosome structure modeling (Carstens *et al.* 2016). Choices like particle pair filtering or interaction computation are left to the user of the library.

**Figure 1.**
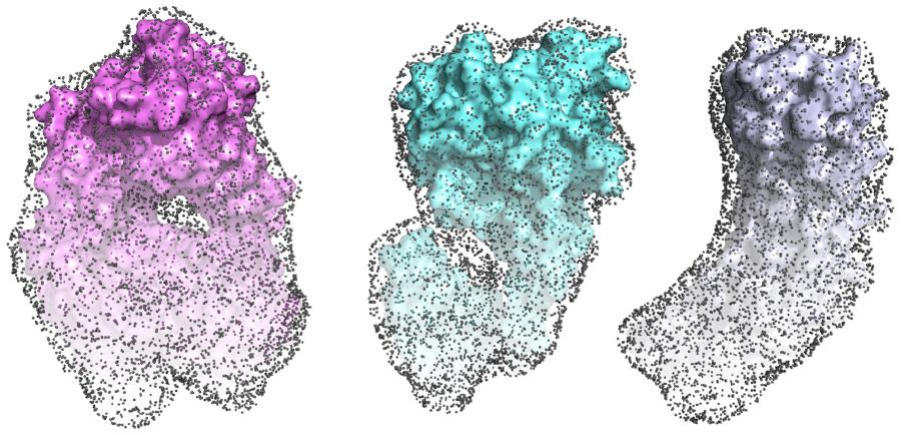
Sampled surfaces of individual chains with PDB IDs 4LU5 IM, 1TMX A, and 4XL5 C (left to right). Per structure, 5000 points (gray) were sampled at 1.05 Å above the surface, $r_{c}=10$. Lower halves of the structures are faded out for visual clarity.

As it happens, zelll is flexible enough to allow for individual neighborhood queries. One practical application of this is (probabilistic) sampling of protein structure surfaces (Fig. 1) which we describe as a case study at a later point.

### 1.1 Basic cell lists

The core idea of the cell lists algorithm consists of dividing a box containing *n* particles of the simulation into cells of edge length $\geq r_{c}$. Then, in O(n) runtime, every particle is assigned to a list corresponding to its surrounding cell.

The enumeration of particle pairs can be seen as computing both all *2-combinations* of each cell list with itself and the *Cartesian product* with its neighboring cell lists, followed by removal of pairs with distances $>r_{c}$. While the Moore neighborhood is a natural choice here, other neighborhood relations facilitate more advanced variants of this algorithm (In ’t Veld *et al.* 2008). Assuming approximately uniformly densely populated cells and *appropriate* (providing constant-time cell index/list lookup, that is) data structures, this is also of linear runtime complexity. Generally, construction follows a pattern:

1. cell index: map particle coordinates to cells
2. cell storage: map cell indices to containers, allocate required memory
3. assign particles to containers by cell index

Details differ vastly between instances of this pattern, e.g. (Allen and Tildesley 2017) describes intertwined linked lists stored in a single contiguous memory allocation, whereas other approaches usually exhibit different allocation strategies, like 2D arrays with maximum neighbor list capacities (Yao *et al.* 2004, Howard *et al.* 2016) or individual contiguous allocations per cell (Gonnet 2012). Many variants (Yao *et al.* 2004, Meloni *et al.* 2007, In ’t Veld *et al.* 2008, Gonnet 2012, Páll and Hess 2013, Li *et al.* 2020, Newcome *et al.* 2023) use both cell and Verlet lists (Verlet 1967). For brevity, we do not discuss additional types of modifications, e.g. concurrency mechanisms, reordering, or stencils. The last step of the aforementioned pattern has the structure of a sorting problem. In fact, our allocation strategy partially reminds of *counting sort* as will become apparent in the next section.

## 2 Implementation details

The previous description of the cell lists algorithm lends itself to an implementation using iterators. While the iterator concept is not unique to the Rust programming language, we benefit from the Rust compiler generally optimizing iterators very well, in addition to encapsulation of mutable state and composability. We focus on design decisions enabling a flexible interface (Listing 1) with good performance.

Listing 1. The typical usage pattern of zelll consists of construction and iteration. Usually, dimension and floating point precision can be inferred from particles. Detailed documentation is available at https://docs.rs/zelll.

1 let cg = CellGrid::new(&particles, CUTOFF);

2 cg.particle_pairs()

3 .filter(|&((_i, p), (_j, q))| {

4 distance(&p, &q) <= CUTOFF

5 })

6 .for_each(|((_i, p), (_j, q))| {

7 dbg!(pair_potential(&p, &q));

8 });

### 2.1 Storage and construction

Like Gonnet (2012), we use contiguous blocks of memory to store particle data for each cell. In contrast, we use a single memory allocation for all cells, as seen in Allen and Tildesley (2017). This is done to leverage spatial and sequential proximity in the input data; neighboring cells contain spatially close particles and in biomolecular structure data, sequentially close particles are often spatially close as well.

However, using a single memory allocation with separate regions for each cell requires us to know the exact cell size beforehand. Note that this approach appears in a range of situations, such as counting sort, jagged arrays, or more generally arena allocation. We count the particles being assigned to each cell by computing *cell indices* from their coordinates (cf. Mattson and Rice 1999) using the shape of the axis-aligned bounding box surrounding the data, similar to how multi-dimensional arrays are typically indexed in linear memory. A hash map stores the occurrences of each cell index value. This does not affect runtime complexity and suffices to assign particle data to cells.

Since there are at most *n* non-empty cells (oftentimes a lot less with sparse or coarse-grained data), we use a hash map to store cell information. This is not a new idea (Bentley *et al.* 1977) that is usually not necessary in common MD settings such as (all-atom) fluid simulations. The whole procedure is illustrated in Algorithm 1.

Algorithm 1. CellGrid construction.

1: **function**  New(*particles*, $r_{c}$)

2:  *indices*  ←  Map(CellIndex rc, *particles*)

3:  *cell_lengths*←  Counts(*indices*)  ▹ this is a hash map

4:  *storage*  ←  Allocate(Length(*indices*))

5:  *cells*  ←  NewHashMap()  ▹ recycles cell_lengths memory

6:  **for all**  i,l∈  KeyValuePairs(*cell_lengths*) **do** 

7:   *cells_i_*  ←  ReserveIn(*storage, l *)  $▹\;cells_{i}\text{ is a slice}$

8:  **end for** 

9:  **for all**  i,p∈  Zip(*indices, particles*) **do** 

10:   Push(*cells_i_, p*)

11:  **end for** 

12:  **return**CellGrid{*indices, storage, cells*}

13: **end function** 

### 2.2 Iteration

zelll realizes particle pair enumeration using existing iterator adapters as sketched out in Algorithm 2.

Algorithm 2. CellGrid iteration illustrated as a generator.

1: **function**  ParticlePairs(*grid*)

2:  **for all**  *i, cell*  ∈  KeyValuePairs(*grid.cells*) **do** 

3:   **for all**  p,q∈ 2Combinations(*cell*) **do** 

4:    **Yield**  p,q

5:   **end for** 

6:   **for all**  j∈  NeighborIndices(*i*) **do** 

7:    **for all**  p,q∈  CartesianProduct(*cell, grid.cells_j_*) **do** 

8:     **Yield**  p,q

9:    **end for** 

10:   **end for** 

11:  **end for** 

12: **end function** 

Here, the effort put into the storage layout pays off; spatial proximity in the input data is reflected by spatial memory locality. Not only do we iterate efficiently on slices, but we can also expect (to some degree) to hit the CPU cache for neighboring slices.

The (*half-*Moore) neighborhood of a cell is determined by means of arithmetic on cell indices followed by hash map lookups. We refrain from using more complex neighborhood shapes as the cost for additional hash map lookups quickly accumulates. Furthermore, the implicit grid boundaries are aperiodic. The iterator interface allows for flexible computation. There are no restrictions on filter and interaction kernel choice (cf. Listing 1).

While parallel iteration is available, we do not provide concurrent collection types ourselves but refer to the Rust crate ecosystem.

## 3 Benchmarks

The primary purpose of the benchmarks provided here is to establish a reasonable runtime estimation one can expect in an unfavorable setting for zelll.

However, additional benchmarks included in the Supplementary Information, available as supplementary data at *Bioinformatics Advances* online show our software is competitive with other published packages.

Therefore, synthetic point cloud data was constructed as follows. A sequence of *n* particle positions is sampled uniformly random in a box of fixed x- and y-axis and varying z-axis, such that a cell grid with $r_{c}=$ 10 Å contains 10 molecules per cube cell on average. This setup ensures

1. surface and volume of the simulation box scaling linearly with *n* to avoid measuring boundary artifacts,
2. maximum number of non-empty neighbor cell hash map lookups during iteration,
3. unstructured benchmark data.

**Figure 2.**
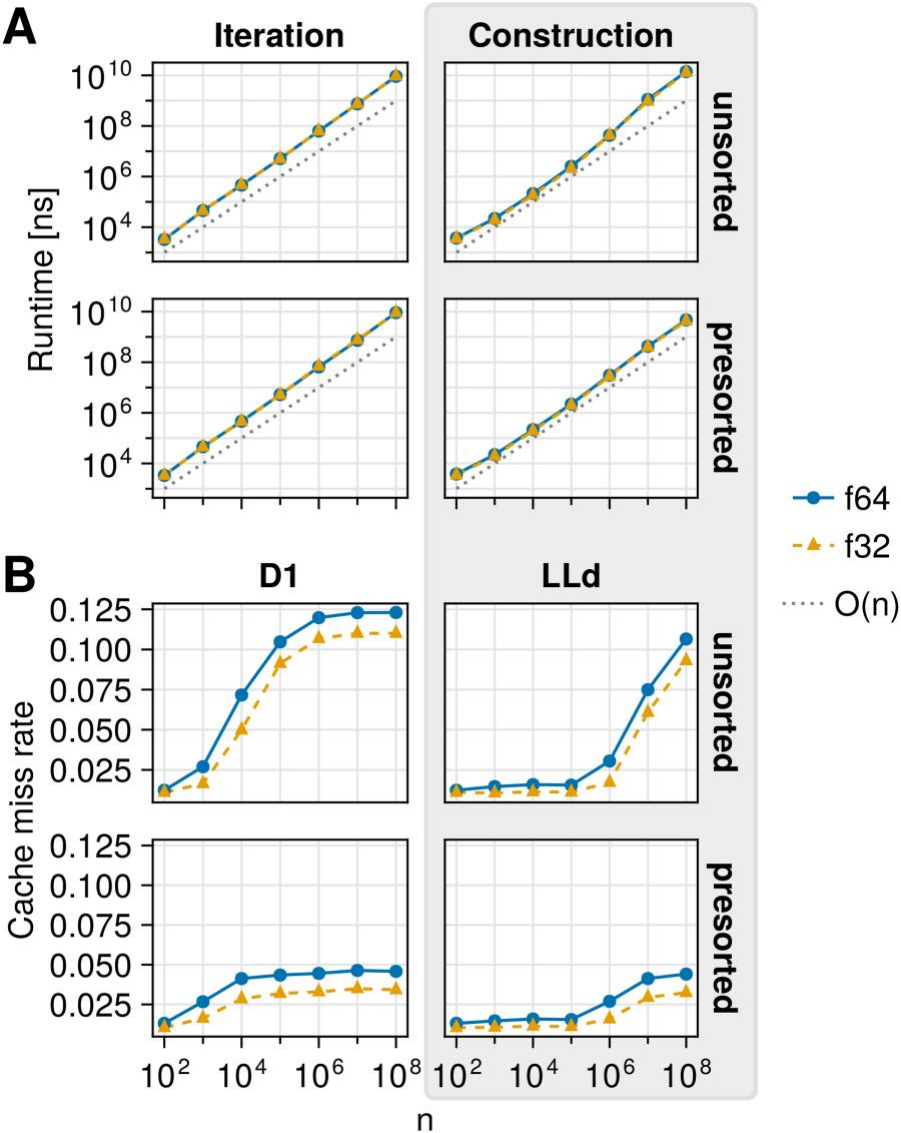
(A) Average runtime measurements for cell grid construction from synthetic data comprised of *n* particles and sequential iteration on an AMD Ryzen 7 3700X processor. Theoretical O(n) time complexity is indicated as a gray dotted line at an arbitrary *y*-axis intercept. (B) Cache miss rates during cell grid construction as measured by Callgrind. D1: 1st-level data cache (256 KiB), LLd: last-level data cache (32 MiB). f32/f64: floating point precision. Presorted data is the same as unsorted data but sorted by its particles’ z-components.

We repeated the benchmark measurements for cell grid construction and iteration with 32 bit and 64 bit floating point precision as well as synthetically structured data (Fig. 2A). The latter is achieved by sorting the sequence of random particle positions by their z-axis components prior to measurement. Iteration encompasses pair enumeration and filtering by squared euclidean distance.

Iteration is largely agnostic to (un)structured data. This is due to the contiguous cell storage internal to zelll. In contrast, cell grid construction is negatively affected by unstructured data.

To investigate deviations from the expected O(n) runtime complexity during construction (Fig. 2A, gray box), we used Callgrind cache simulations (Weidendorfer *et al.* 2004, Nethercote and Seward 2007). With two simulated cache levels, the deviations can be attributed in large part to increased last-level cache (**LLd**) miss rates (Fig. 2B, gray box). First-level data cache (**D1**) misses accumulate at smaller *n* but are not expected to contribute as much as higher cache levels. Overall, cache miss rates tend to saturate and cell grid construction benefits from both lower floating point precision and structured data.

Parallel iteration is subject to overhead from setting up the necessary threads. This is a dominant factor for small $n\approx 10^{4}$ (see Supplementary Information, available as supplementary data at *Bioinformatics Advances* online for details).

## 4 Case study

zelll can be used in similar contexts as other partitioning data structures. Notably, this includes the representation of biomolecular structures using signed distance functions and relatives (Sverrisson *et al.* 2021, Scott *et al.* 2025). Here, we relax the *signedness* requirement and focus on a specific *smooth* distance function instead.

Given a set $P\subset R^{3}$ of all atoms in a protein structure, the neighborhood within a cutoff radius $r_{c}$ of a query location $x\in R^{3}$ is $N_{x}^{r_{c}}:=\{p\in P|r_{c}\geq ∥x-p∥\}$. Because zelll supports querying $N_{x}^{r_{c}}$, we can construct an approximation of the *exact* smooth distance function used in dMaSIF (Sverrisson *et al.* 2021) (see also Supplementary Information, available as supplementary data at *Bioinformatics Advances* online):


(1) $${\tilde{\text{SDF}}}_{P}(x)=-\tilde{\sigma }(x)\cdot \;\text{log}\;\sum_{p\in N_{x}^{r_{c}}}\text{exp}(-∥x-p∥/\sigma_{p})$$

where $\tilde{\sigma }(x)=\sum_{p\in N_{x}^{r_{c}}}\;\text{exp}\;(-∥x-p∥)\sigma_{p}/\sum_{p\in N_{x}^{r_{c}}}\text{exp}\;(-∥x-p∥)$ is a similar approximation of the average atom radius in a neighborhood of x, with $\sigma_{p}$ denoting the atom radius of individual atoms p.

The surface of a protein structure is then understood as a fixed level set of the *approximate* smooth distance function. Instead of minimizing the squared loss function, we sample positions of a single particle in a harmonic potential centered at the 1.05 Å level set surface using the No-U-Turn sampler (NUTS) (Homan and Gelman 2014). This requires a gradient which is easily obtained by automatic differentiation (Rehner and Bauer 2021) of the harmonic potential composed with the approximate smooth distance function. With ${\tilde{\text{SDF}}}_{P}$ being an approximation, there is a trade-off between the error it introduces and computational cost. Individual queries take a few microseconds, depending on $r_{c}$ (cf. Supplementary Information, available as supplementary data at *Bioinformatics Advances* online). Aside from computational overhead, zelll itself is agnostic to querying these surfaces individually or together. Realistically, sampling on disjoint surfaces using NUTS would also require sampling multiple particles simultaneously.

While this procedure achieves good surface coverage in practice (Fig. 1), we emphasize that it is not intended for computer graphics use cases like real-time rendering. Its probabilistic nature instead alludes to Bayesian approaches taken in biomolecular structure modeling (Habeck 2023). Here, the surface of a protein structure represents a prior distribution for which we described a procedure to sample from. Potential applications depend on the determination of a suitable likelihood model for surface features. In the context of protein docking, such a model could be informed by known protein complexes or experimental data (Mandell *et al.* 2005, Koukos and Bonvin 2020) to sample potential protein interaction sites. Our case study offers an instructional entry point to formulate similar problems in a probabilistic way and highlights the modularity facilitated by zelll combined with a sampler from a third-party package. We see value in applying zelll to expand on previous work taking a Bayesian perspective on chromosome structure modeling (Carstens *et al.* 2016).

## 5 Summary

We introduce an implementation of the cell lists algorithm for the Rust programming language with Python bindings for its core functionality. We describe its characteristics focusing on its intended use with sparse or coarse-grained biomolecular structure data. Its application is illustrated by a sampling-based case study with aspects from our ongoing research efforts.

## Supplementary Material

vbaf330_Supplementary_Data

## Data Availability

Code used to generate benchmark data is available with the library source code and is archived on Zenodo: https://doi.org/10.5281/zenodo.18183795.
