# Supplementary material for: zelll: a fast, framework-free, and flexible implementation of the cell lists algorithm for the Rust programming language
Source: Bioinform Adv. 2026 Jan 2;6(1):vbaf330. doi: 10.1093/bioadv/vbaf330 (PMC12910374; doi:10.1093/bioadv/vbaf330)
Supplement: vbaf330_Supplementary_Data [file vbaf330_supplementary_data.pdf]

Supplementary Information

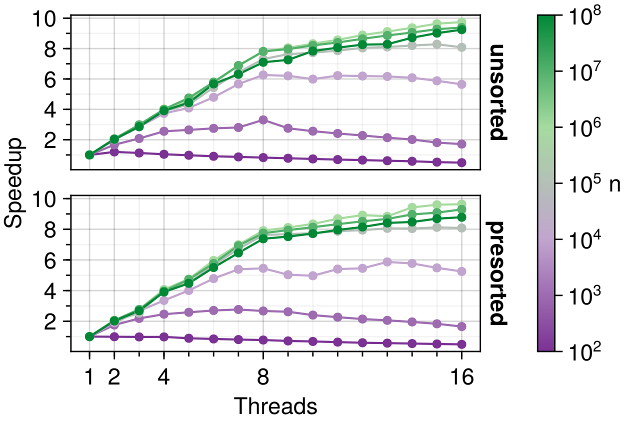

Fig. S1: Parallel iteration speedup by number of (software) threads i on a 8-core processor with two logical CPUs per core. The benchmark setup is restricted to `f64` particle data but follows the main text otherwise. Speedup is defined as t_{seq}/t_i. Reduced speedup for $i > 8$ indicates resources shared by logical CPUs being the primary bottleneck.
Without CPU affinity-aware scheduling, one thread per core achieves better parallel efficiency than simultaneous multithreading; for large n it is close to ideal due to the cell-wise data layout.

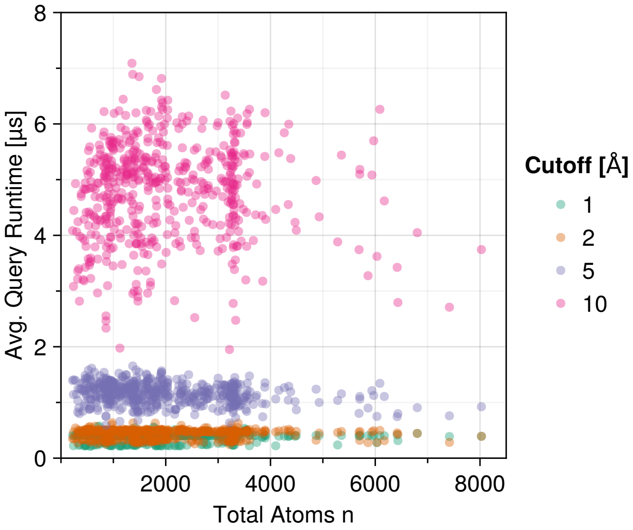

Fig. S2: Average runtime of $\widetilde{\mathrm{SDF}}_P$ queries by number of atoms in protein structures, including evaluation of $\nabla\widetilde{\mathrm{SDF}}_P$ via automatic differentiation. Unbound protein structures from the docking benchmark version 5 (Vreven *et al.*, 2015) were used for measurements. Runtime is accumulated over 64^3 queries covering each structure's axis-aligned bounding box in a grid followed by normalization (cf. Scott *et al.* (2025)).

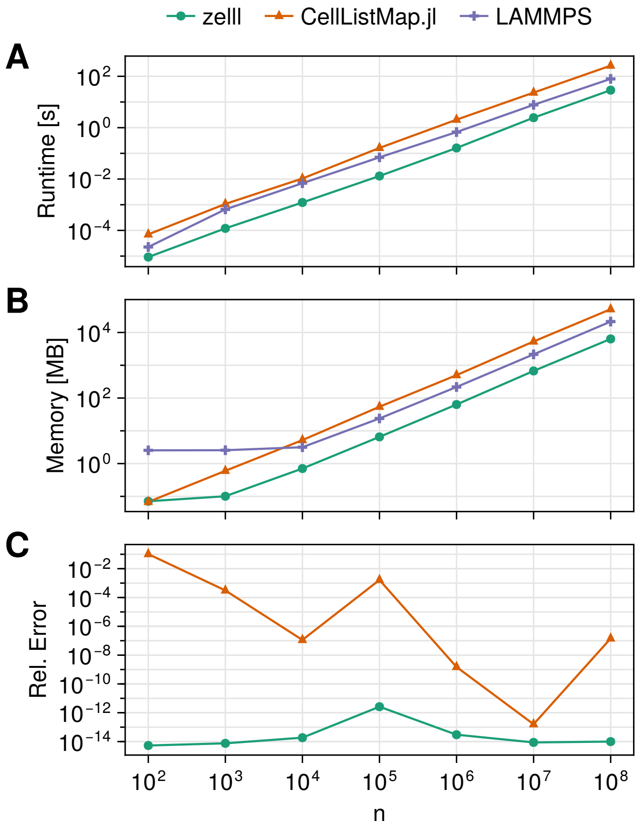

Fig. S3: Comparison of `zelll` with *CellListMap.jl* and *LAMMPS* respectively. Input data is identical for all of the shown software packages and constructed as described in the main text. Measurements comprise (sequential) data structure construction, iteration and computation of pairwise dimensionless Lennard-Jones potentials accumulated into the average total energy per particle of the synthetic system. **A:** Average runtime, **B:** allocated memory, **C:** relative error of the computed total energies with respect to the value computed by *LAMMPS*.

Performance comparisons with other software packages implementing cell list variants (Figure S3) indicate that `zelll` is fit for practical use. Compared to *CellListmap.jl* (Martínez, 2022), `zelll` performs approximately ten times better in this setting. However, fair benchmarks are usually difficult to achieve, as is the case here.

LAMMPS (Thompson *et al.*, 2022) self-reports diagnostical data and does provide more functionality than `zelll`. *CellListMap.jl* is written in Julia and relies on automatic garbage collection. Characteristics like these can affect the direct interpretability of benchmarks, especially across different programming languages. Nevertheless, the benchmarked packages differ not more than one order of magnitude in performance. Depending on the specific application intended, they can be used effectively for scientific computing.

Like our library, benchmark code is available at `https://github.com/microscopic-image-analysis/zelll`.

Listing S1. An excerpt of a working Python implementation of Equation 1 in the main text closely resembling the Rust code written for the case study described in the main text. Both implementations are available in the `zelll` repository. Automatic differentiation via (Rehner and Bauer, 2021) is also shown.

```python
def _sdf(pos, neighbors):
   scaled_exp_dists = 0.0
   atom_radii = 0.0
   total_exp_dists = 0.0

   for radius, coords in neighbors:
      dist = np.linalg.norm(np.asarray(pos) -
         np.asarray(coords))
      if dist.value != 0.0:
         scaled_exp_dists += np.exp(-dist / radius)
         atom_radii += np.exp(-dist) * radius
         total_exp_dists += np.exp(-dist)
      else:
         scaled_exp_dists += 1.0
         atom_radii += radius
         total_exp_dists += 1.0

   sigma = atom_radii / total_exp_dists
   return -sigma * np.log(scaled_exp_dists)

# queries neighborhood and sets up data
# for SDF and gradient evaluation
def eval_sdf(pos, cg: CellGrid, radii=None):
   # here, ‘zelll‘ is used
   neighbors = cg.neighbors(pos);
   if not neighbors:
      return -np.inf, [-np.inf, -np.inf, -np.inf]

   if self.radii:
      neighbors = [(radii[i].value, coords)
         for i, coords in neighbors]
   else:
      neighbors = [(1.0, coords)
         for i, coords in neighbors]
   # ‘neighbors’ is treated as a constant
   return gradient(lambda x: _sdf(x, neighbors), pos)
```

References

Martínez, L. (2022). CellListMap.jl: Efficient and customizable cell list implementation for calculation of pairwise particle properties within a cutoff. *Computer Physics Communications*, **279**, 108452.

Rehner, P. and Bauer, G. (2021). Application of Generalized (Hyper-) Dual Numbers in Equation of State Modeling. *Frontiers in Chemical Engineering*, **3**.

Scott, C. B. *et al.* (2025). Implicitly and Differentiably Representing Protein Surfaces and Interfaces. arXiv:2508.11641 [q-bio].

Thompson, A. P. *et al.* (2022). LAMMPS - a flexible simulation tool for particle-based materials modeling at the atomic, meso, and continuum scales. *Computer Physics Communications*, **271**, 108171.

Vreven, T. *et al.* (2015). Updates to the integrated protein-protein interaction benchmarks: Docking benchmark version 5 and affinity benchmark version 2. *Journal of molecular biology*, **427**(19), 3031–3041.
